# Rv2969c, essential for optimal growth in *Mycobacterium tuberculosis*, is a DsbA-like enzyme that interacts with VKOR-derived peptides and has atypical features of DsbA-like disulfide oxidases

**DOI:** 10.1107/S0907444913017800

**Published:** 2013-09-20

**Authors:** Lakshmanane Premkumar, Begoña Heras, Wilko Duprez, Patricia Walden, Maria Halili, Fabian Kurth, David P. Fairlie, Jennifer L. Martin

**Affiliations:** a Institute for Molecular Bioscience, Division of Chemistry and Structural Biology, University of Queensland, St Lucia, QLD 4067, Australia

**Keywords:** DsbA, VKOR, DsbB, antibacterial target, oxidative folding, virulence, thioredoxin

## Abstract

The bacterial disulfide machinery is an attractive molecular target for developing new antibacterials because it is required for the production of multiple virulence factors. The archetypal disulfide oxidase proteins in *Escherichia coli* (Ec) are DsbA and DsbB, which together form a functional unit: DsbA introduces disulfides into folding proteins and DsbB re­oxidizes DsbA to maintain it in the active form. In *Mycobacterium tuberculosis* (Mtb), no DsbB homologue is encoded but a functionally similar but structurally divergent protein, MtbVKOR, has been identified. Here, the Mtb protein Rv2969c is investigated and it is shown that it is the DsbA-like partner protein of MtbVKOR. It is found that it has the characteristic redox features of a DsbA-like protein: a highly acidic catalytic cysteine, a highly oxidizing potential and a destabilizing active-site disulfide bond. Rv2969c also has peptide-oxidizing activity and recognizes peptide segments derived from the periplasmic loops of MtbVKOR. Unlike the archetypal EcDsbA enzyme, Rv2969c has little or no activity in disulfide-reducing and disulfide-isomerase assays. The crystal structure of Rv2969c reveals a canonical DsbA fold comprising a thioredoxin domain with an embedded helical domain. However, Rv2969c diverges considerably from other DsbAs, including having an additional C-terminal helix (H8) that may restrain the mobility of the catalytic helix H1. The enzyme is also characterized by a very shallow hydrophobic binding surface and a negative electrostatic surface potential surrounding the catalytic cysteine. The structure of Rv2969c was also used to model the structure of a paralogous DsbA-like domain of the Ser/Thr protein kinase PknE. Together, these results show that Rv2969c is a DsbA-like protein with unique properties and a limited substrate-binding specificity.

## Abstract 2

The gene product of *M. tuberculosis* Rv2969c is shown to be a disulfide oxidase enzyme that has a canonical DsbA-like fold with novel structural and functional characteristics.

## 1. Introduction

The devastating disease tuberculosis (TB) caused by the bacterium *Mycobacterium tuberculosis* (Mtb) is responsible for approximately two million deaths annually. The loss of effectiveness of the only available TB vaccine, Bacillus Calmette–Guérin (BCG), for people of economically productive age (15–59 years) has created an enormous drain on the world economy (World Health Organization, 2011 ▶). A major hurdle to eradicating TB is the requirement for multi-antibiotic therapy administered over a period of six to nine months (Connolly *et al.*, 2007 ▶). Multidrug-resistant (MDR) and extensively drug-resistant (XDR) Mtb strains have evolved for which therapeutic options are limited to toxic and expensive second-line and third-line reserved drugs (Phillips, 2013 ▶). Aside from MDR and XDR strains, Mtb has evolved mechanisms to succeed as an intracellular pathogen by manipulating the host immune signalling responses (Kumar & Narayanan, 2012 ▶; Pieters, 2008 ▶). Mtb virulence factors can avert host-cell apoptosis, phagosome maturation and auto­phagy to gain intracellular persistence, hence restricting the adaptive immune response (Vergne *et al.*, 2004 ▶; Welin *et al.*, 2011 ▶; Songane *et al.*, 2012 ▶; Kim *et al.*, 2012 ▶). Considering that one third of the world’s population is infected with TB, there is a compelling need to identify new molecular targets for the development of antituberculosis drugs as alternatives or additions to the current broad-spectrum traditional antibiotics (Raman *et al.*, 2012 ▶; Murillo *et al.*, 2007 ▶; Lou & Zhang, 2010 ▶).

Mtb cell-surface and secretory proteins are known virulence components for mycobacterial growth during infection and for continued survival in host cells (Zhou *et al.*, 2010 ▶; Scherr *et al.*, 2007 ▶; Feltcher *et al.*, 2010 ▶; DiGiuseppe Champion & Cox, 2007 ▶). Consequently, antivirulence approaches targeting individual proteins responsible for mycobacterial virulence are being intensively investigated (Feltcher *et al.*, 2010 ▶). While these approaches offer some advantages, there is also a case to be made for a therapeutic strategy that interferes with the early stages of Mtb infection and that affects multiple virulence targets.

Periplasmic disulfide-bond forming (Dsb) enzymes catalyze the oxidative folding and maturation of many toxins and surface proteins required for virulence in a range of pathogenic bacteria (Heras *et al.*, 2009 ▶). Typically, Dsb proteins include a soluble thioredoxin (Trx) fold protein DsbA, which introduces disulfide bonds into newly translocated proteins in the periplasm (Heras *et al.*, 2009 ▶; Kadokura *et al.*, 2003 ▶; Inaba, 2009 ▶), and its cognate inner membrane partner protein DsbB that keeps DsbA in the functionally active oxidized form (Inaba & Ito, 2008 ▶; Inaba *et al.*, 2006 ▶). Deletion of DsbA or DsbB has pleiotropic effects on virulence-factor production and in some cases can provide a marginal increase in sensitivity to conventional antibiotics (Hayashi *et al.*, 2000 ▶). Both DsbA and DsbB have therefore been identified as potential antibacterial targets to combat virulence of pathogenic bacteria (Heras *et al.*, 2009 ▶; Yu, 1998 ▶). DsbA and DsbB enzymes are not always conserved across bacterial species, although the oxidative-folding pathway seems to be widely required for bacterial virulence (Dutton *et al.*, 2008 ▶).

Approximately 60% of Mtb exported proteins are estimated to require a disulfide bond for activity, stability and protease resistance (Chim *et al.*, 2011 ▶; Goulding *et al.*, 2004 ▶). However, the mycobacterial genome is deficient in a DsbB sequence homologue. Moreover, a DsbA-like protein responsible for disulfide oxidase activity has not been confirmed in *Mycobacterium*, although the Mtb gene product of Rv2969c (annotated as uncharacterized) has been identified as a potential candidate DsbA (Wang *et al.*, 2011 ▶). DsbA enzymes typically comprise two domains: a noncontiguous Trx domain, which has a characteristic redox-active C*XX*C motif and a *cis*-Pro motif, and a helical domain inserted into the Trx domain. Aside from the Trx domain motifs, sequence conservation among DsbA enzymes is very low. The Mtb genome contains two genes encoding remote DsbA homologues, Rv2969c and a transmembrane serine/threonine protein kinase protein incorporating a DsbA-like domain (PknE; Rv1743; I6YBI4).

A gene adjacent to the *dsbA*-like Rv2969c gene encodes a mammalian homologue of vitamin K epoxide reductase (VKOR; Rv2968c; Dutton *et al.*, 2008 ▶). In humans, deficiency of VKOR or administration of its antagonist warfarin inhibits reduction of vitamin K 2,3-epoxide that is otherwise required for blood clotting. In an elegant series of studies, the Beckwith group proposed that MtbVKOR may represent the functional equivalent of *E. coli* DsbB (EcDsbB) and showed that MtbVKOR rescues motility of *E. coli dsbB* null cells (Dutton *et al.*, 2008 ▶; Wang *et al.*, 2011 ▶). This notion was further supported by the observation that some plants and bacteria encode a Trx-VKOR fusion protein. A crystal structure of a *Cyanobacterium* Trx-VKOR fusion confirmed that VKOR and DsbB are functionally similar but structurally divergent (Li *et al.*, 2010 ▶).

Throughout *Mycobacterium*, Rv2969c and VKOR are genetically linked. This may indicate that they are functionally related genes. Genome-wide transposon-insertion mutagenesis studies showed that these two genes are essential for the optimal growth of Mtb (Sassetti *et al.*, 2003 ▶). This is in agreement with the observation that deletion of VKOR in *M. smegmatis* confers severe growth defects (Wang *et al.*, 2011 ▶). Moreover, the anticoagulant medication warfarin can inhibit growth of Mtb in a VKOR-dependent manner (Dutton *et al.*, 2010 ▶).

Here, we present a detailed structural and functional characterization of Rv2969c, the remote DsbA homologue encoded by Mtb (which we refer to as MtbDsbA). The structure of MtbDsbA reveals a number of modifications to the archetypal EcDsbA structure and to the binding surface surrounding the active-site cysteine. The *in vitro* activity of MtbDsbA indicates that it is a mycobacterial disulfide oxidase and its ability to bind peptides derived from MtbVKOR supports the notion that MtbDsbA and MtbVKOR form a functional redox pair. MtbDsbA may therefore represent an important target for the development of antituberculosis drugs that block oxidative folding of exported Mtb proteins necessary for mycobacterial infection and survival within host macrophages. The structure that we report may serve as a starting point for rational drug design towards this end.

## 2. Experimental procedures

### 2.1. Cloning, expression and protein production

The N-terminal region of MtbDsbA is predicted to be a secretion signal (*SignalP*; Emanuelsson *et al.*, 2007 ▶) or transmembrane anchor (TMHMM; Krogh *et al.*, 2001 ▶). A codon-optimized synthetic gene corresponding to the soluble MtbDsbA enzyme lacking this region (*M. tuberculosis* H37Rv, residues 46–255) was inserted into the bacterial expression vector pMCSG7 by ligation-independent cloning (Eschenfeldt *et al.*, 2009 ▶). An amino-terminal His_6_-tagged MtbDsbA was expressed in *E. coli* BL21(DE3) cells using auto-induction medium (Studier, 2005 ▶). Protein was purified using TALON cobalt resin (Clontech) and the His_6_ tag was removed by TEV protease leaving three vector-derived residues (Ser-Asn-Ala) at the N-terminus. For crystallization experiments, the protein was incubated with 100 m*M* oxidized glutathione to generate the oxidized enzyme, prior to final purification on a Superdex 75 gel-filtration column (GE Healthcare). Site-directed mutagenesis was performed using the QuikChange method and the mutation was confirmed by DNA sequencing. A noncatalytic double cysteine mutant MtbDsbA^m^ (Cys140Ala, Cys192Ala) and an active-site single cysteine mutant MtbDsbA (Cys92Ala) were expressed and purified in the same way as for wild-type MtbDsbA.

### 2.2. Crystallization and diffraction data collection

MtbDsbA crystals were grown by the hanging-drop vapour-diffusion method at 293 K; drops were set up using a Mosquito crystallization robot (TTP Labtech) and were incubated and imaged in a RockImager 1000 (Formulatrix). 250 nl MtbDsbA solution concentrated to 55 mg ml^−1^ in 25 m*M* HEPES pH 7.4, 100 m*M* NaCl was mixed with 250 nl reservoir solution consisting of 2.4 *M* sodium malonate pH 5.5, 3.7% 1,4-dioxane, 0.08% polyvinylpyrrolidone. Crystals grew as long thin rods (∼30 × 500 µm) in 3–4 weeks and were flash-cooled in liquid nitrogen after brief rinsing in 3.4 *M* sodium malonate pH 5.5. Diffraction data were collected on the MX2 beamline at the Australian Synchrotron at a wavelength of 0.9537 Å and were recorded using an ADSC Quantum 315r detector controlled by *Blu-Ice* (McPhillips *et al.*, 2002 ▶). Data were integrated in *XDS* (Kabsch, 2010 ▶), space-group possibilities were analyzed using *POINTLESS* (Evans, 2006 ▶) and data were scaled in *SCALA* from the *CCP*4 suite (Winn *et al.*, 2011 ▶).

### 2.3. Structure determination and refinement

Molecular-replacement (MR) searches for MtbDsbA with *Phaser* (McCoy *et al.*, 2007 ▶) using intact DsbA templates (less than 21% sequence identity) were unsuccessful. Regions contributing to DsbA structural divergence were identified by superposition of DsbA structures currently available in the PDB and the structurally divergent regions were trimmed to generate a collection of DsbA ‘Poly Ser’ MR templates (nonglycine and non-alanine residues that were not conserved were changed to serine) for MR trials in *Phaser*. A Poly Ser conserved structural core search model derived from *Staphylo­coccus aureus* DsbA (SaDsbA) (PDB entry 3bci; Heras *et al.*, 2008 ▶) gave an MR solution (TFZ = 8.0) with two copies in the asymmetric unit. This trimmed MR search model provided 39% coverage with 48% identity to the MtbDsbA sequence (Supplementary Fig. S1^1^). Iterative manual building, density modification and refinement in *PHENIX* (Adams *et al.*, 2010 ▶) and *Coot* (Emsley & Cowtan, 2004 ▶) allowed tracing of almost the entire MtbDsbA sequence. Final refinement cycles involving TLS were carried out in *phenix.refine*. Electron density was absent for the three vector-derived residues and the N-­terminal 11 residues of the MtbDsbA construct in both molecules in the asymmetric unit. At the interface of the two MtbDsbA molecules in the asymmetric unit, two additional regions of electron density were observed in the difference Fourier map contoured at 3.0σ. These were interpreted as 1,4-­dioxane and HEPES, both of which were present in the crystallization condition. A summary of the data-processing and refinement statistics is presented in Table 1 ▶. The stereochemical quality of the final model was assessed using *AutoDep Input Tool* (Yang *et al.*, 2004 ▶), *MolProbity* (Chen *et al.*, 2010 ▶) and *SFCHECK* (Vaguine *et al.*, 1999 ▶). The coordinates and structure factors have been deposited in the Protein Data Bank (PDB entry 4k6x).

### 2.4. Isothermal titration calorimetry (ITC)

Experiments were carried out on a Microcal Auto-iTC200 (GE Healthcare). Peptides with N-terminal acetylation and C-­terminal amidation were chemically synthesized manually by classic solid-phase peptide synthesis using rink amide MBHA resin (ChemImpex International, loading 0.65 mmol g^−1^) and Fmoc-protected amino acids (ChemImpex International). Peptides were purified by reversed-phase HPLC (C18, Phenomenex) and characterized by retention times, electrospray mass spectrometry and ^1^H NMR spectroscopy. Each ITC titration involved an initial 0.5 µl injection (not included in the subsequent analysis) followed by 19 injections of 2 µl of 2–4 m*M* peptide into cells containing 100 µ*M* MtbDsbA in 25 m*M* HEPES pH 7.4, 100 m*M* NaCl at 298 K. Thermodynamic parameters were obtained from nonlinear curve fitting using a single-site binding mode in *Origin* 7.0 (SR4 v7.0552 beta, Microcal).

### 2.5. Thermal stability measurement

Temperature-induced unfolding of MtbDsbA was recorded by far-UV circular dichroism (CD), with the temperature increasing from 298 to 368 K at a rate of 1 K min^−1^ using a Jasco J-810 spectropolarimeter. The normalized CD signal for oxidized (219 nm) and reduced (220.5 nm) MtbDsbA in 100 m*M* phosphate buffer, 1 m*M* ethylenediaminetetraacetic acid (EDTA) pH 7.0 was fitted to a two-state unfolding model as described previously (Kurz *et al.*, 2009 ▶). The redox status of the oxidized and reduced protein preparation with oxidized glutathione and DTT (dithiothreitol), respectively, was verified with Ellman’s reagent.

### 2.6. Insulin-reduction assay

The catalytic ability of MtbDsbA to reduce insulin in the presence of DTT was monitored by the increase in absorbance at 650 nm over 80 min as described previously (Heras *et al.*, 2008 ▶). Briefly, the reaction mixture contained 131 µ*M* insulin and 10 µ*M* of oxidized MtbDsbA or EcDsbA in 100 m*M* phosphate buffer pH 7.0, 2 m*M* EDTA. The reaction was initiated by adding DTT to a final concentration of 0.35 m*M*. The noncatalyzed aggregation of insulin by DTT was monitored in a control reaction without enzyme.

### 2.7. Isomerization of scrambled RNaseA

*In vitro* disulfide isomerase activity of MtbDsbA, EcDsbC and EcDsbA was monitored using a scrambled RNaseA refolding assay (Hillson *et al.*, 1984 ▶). Scrambled RNaseA was produced as previously described (Heras *et al.*, 2008 ▶). Isomerization of scrambled RNaseA (40 µ*M*) was conducted in 100 m*M* sodium phosphate buffer containing 1 m*M* EDTA pH 7.0, 10 m*M* DTT and 10 µ*M* DsbA or DsbC at 298 K (750 µl). At various time intervals, 50 µl of refolded RNase sample was taken to monitor the hydrolysis activity of cytidine 3′,5′-cyclic monophosphate (3 m*M*) using a BioTek H1 plate reader at 296 nm. Native RNaseA and scrambled RNaseA in the absence of DsbA or DsbC served as positive and negative controls, respectively.

### 2.8. Ubiquinone-reduction assay

DsbB-catalyzed oxidation of DsbA can be monitored by following the decrease in ubiquinone absorbance at 275 nm (Bader *et al.*, 2000 ▶). The reaction mixture (70 µl) contained 30 µ*M* reduced MtbDsbA or EcDsbA, 30 µ*M* ubiquinone-1 (UQ1) in 50 m*M* Tris pH 7.0, 100 m*M* NaCl and 0.01% *n*-­dodecyl β-d-maltoside. The decrease in absorbance was recorded for 3 min at 303 K after addition of 57 n*M* EcDsbB using a Varian Cary 50 UV–visible spectrophotometer.

### 2.9. Determination of redox potential

Oxidized MtbDsbA^m^ (2 µ*M*) was incubated in 200 µl degassed buffer consisting of 100 m*M* phosphate buffer, 1 m*M* EDTA pH 7.0, 1 m*M* oxidized glutathione (GSSG) for 1 h at room temperature. The protein was then incubated in buffers with a range of reduced glutathione (GSH) concentrations (0.01 µ*M*–1 m*M*) for 24 h at 298 K (10 µl per reaction containing 2 µ*M* protein concentration). After incubation, the reactions were stopped with 10% trichloroacetic acid and the precipitated protein pellets were collected by centrifugation at 14 000 rev min^−1^ for 10 min at 277 K. The pellets were washed with 100% ice-cold acetone and dissolved in buffer consisting of 50 m*M* Tris pH 7.0, 1% SDS, 4 m*M* 4-acetamido-4′-male­imidylstilbene-2,2′-disulfonate (AMS) to label the free thiols. Separation of reduced and oxidized forms was performed on a NuPAGE 12% bis-tris gel (1.0 mm thick, 12 well; Invitrogen, Australia). The gel was stained with Coomassie, scanned (Perfection V700 Scanner, Epson) and intensities of the reduced protein were analyzed using *ImageJ* v.1.42q (Schneider *et al.*, 2012 ▶). The fraction of the reduced protein was plotted against the ratio [GSH]^2^/[GSSG] and the equilibrium constant *K*_eq_ was calculated using the binding equation *Y* = ([GSH]^2^/[GSSH])/{*K*_eq_ + ([GSH]^2^/[GSSH])}, where *Y* is the fraction of reduced protein at equilibrium. The redox potential was calculated using the Nernst equation *E* ^0′^ = *E* ^0′^_GSH/GSSG_ − (*RT*/*nF*)ln*K*_eq_, where *E* ^0′^_GSH/GSSG_ is the standard potential of −240 mV (Gilbert, 1995 ▶), *R* is the universal gas constant 8.314 J K^−1^ mol^−1^, *T* is the absolute temperature in K, *n* is the number of electrons transferred, *F* is the Faraday constant 9.648 × 10^4^ C mol^−1^ and *K*_eq_ is the equilibrium constant.

### 2.10. Determination of p*K*_a_

The pH-dependent UV absorbance of the nucleophilic cysteine thiol was followed at 240 nm (Nelson & Creighton, 1994 ▶). Measurements of oxidized or reduced MtbDsbA^m^ (lacking the second disulfide) of 24 or 16 µ*M* in a total volume of 200 µl buffer comprising 10 m*M* dipotassium phosphate, 10 m*M* monopotassium phosphate, 10 m*M* sodium citrate, 10 m*M* Tris, 1 m*M* EDTA, 200 m*M* KCl were performed at pH values ranging from 2.2 to 7.5 using Greiner 96-well UV-Star microplates. The redox status of the protein preparation was verified with Ellman’s reagent. The absorbance at 240 and 280 nm was recorded on a BioTek Synergy H1 microplate reader and corrected for blank absorbance. The pH-dependent thiolate-specific absorbance signal [(*A*_240_/*A*_280_)_reduced_/(*A*_240_/*A*_280_)_oxidized_] was fitted to the Henderson–Hasselbach equation as described previously (Kurz *et al.*, 2009 ▶).

### 2.11. Complementation assay

Complementation of EcDsbA by MtbDsbA was monitored in a cell-swarming assay as described previously (Kurz *et al.*, 2009 ▶). A chimeric gene encoding an EcDsbA signal peptide fused to the mature form of MtbDsbA was cloned into the arabinose-inducible pBAD33 vector (Guzman *et al.*, 1995 ▶). A nonmotile *E. coli* *dsbA* ^−^ mutant (JCB817) strain transformed with this chimera construct was stabbed into a minimal agar (M63) plate supplemented with 40 mg ml^−1^ of each amino acid and 1 mg ml^−1^ arabinose. The swarming of *E. coli* cells was analyzed after incubating the plate for 4–6 h at 303 K. JCB817 cells transformed with pBAD33::EcDsbA were used as a positive control. A nonmotile *dsbA* ^−^/*dsbB* ^−^ double-mutant strain (JCB818) served as a negative control. Plates without arabinose were used to monitor background complementation.

### 2.12. Peptide-oxidation assay

A synthetic peptide substrate CQQGFDGTQNSCK with a europium DOTA (1,4,7,10-tetraazacyclododecane-1,4,7,10-tetraacetic acid europium) chelate at the N-terminus and a methylcoumarin amide at the ∊-amino group of the lysine was obtained from Anaspec, USA (for further details of the peptide, see Vivian *et al.*, 2009 ▶). Disulfide-bond formation of the peptide was fluorometrically followed using time-resolved fluorescence with excitation at 340 nm and emission at 615 nm, a delay of 150 µs and a reading time of 100 µs in a Synergy H1 multimode plate reader (BioTek, USA). The assay was performed in a 384-well white plate (PerkinElmer OptiPlate) containing EcDsbA or MtbDsbA and 2 m*M* GSSG in 50 m*M* MES, 50 m*M* NaCl, 2 m*M* EDTA at pH 5.5. The reaction was initiated by the addition of substrate peptide at 4–8 µ*M* in a total volume of 50 µl. The reaction was performed in triplicate and repeated at various concentrations of EcDsbA or MtbDsbA. The reaction in the absence of enzyme was used as a control.

## 3. Results

### 3.1. Putative Mtb DsbA-like proteins

To identify potential Mtb DsbA-like oxidases, we searched the genome of the virulent strain Mtb H37Rv by using keyword and *BLASTP* searches. 15 Trx-related gene products were identified (Table 2 ▶) and nine of these were eliminated as potential DsbA-like proteins as they are likely to be located in the cytoplasm (on the basis of literature reports, the absence of sorting signal/transmembrane region and sequence-to-structure relationships). Of the remaining six predicted to be extracytoplasmic Trx-related proteins, only the sequences of hypothetical Rv2969c and the DsbA-like domain of PknE (Rv1743) remotely resembled DsbA homologues. The sequence identity of the nearest structurally characterized neighbour, *Bacillus subtilis* DsbA (BsDsbA), is less than 19% (by sequence-based alignment) to these two Mtb DsbA paralogues. Rv2969c and Rv1743 share ∼50% sequence similarity (31% identity) and both contain four conserved cysteine residues. The sequence positions of two of the four cysteines are similar to that of the characteristic redox-active C*XX*C motif of the Trx fold, whereas the other two cysteines are consistent with the structural (noncatalytic) cysteines found in *Wolbachia pipientis* α-DsbA1 (Wpα-DsbA1; Kurz *et al.*, 2009 ▶).

The presence of a predicted sorting signal or transmembrane helix in Rv2969c (currently annotated as uncharacterized) indicates it is either secreted into, or membrane-anchored with the catalytic domain facing, the recently characterized mycobacterial periplasm (Patarroyo *et al.*, 2008 ▶; Sani *et al.*, 2010 ▶) and may therefore represent a potential candidate for a DsbA-like protein. Hereafter, we refer to it as MtbDsbA. Its paralogue PknE is a single-pass transmembrane Ser/Thr protein kinase that comprises an extracytoplasmic DsbA-like domain and a cytoplasmic kinase domain (Gay *et al.*, 2006 ▶). PknE has been shown to regulate cellular events in response to host-mediated apoptotic stimuli such as nitric oxide (Jayakumar *et al.*, 2008 ▶). As such, PknE appears to promote the survival of Mtb in the latent state by suppressing host apoptosis (Kumar & Narayanan, 2012 ▶).

### 3.2. MtbDsbA is highly oxidizing

**Figure 1:**
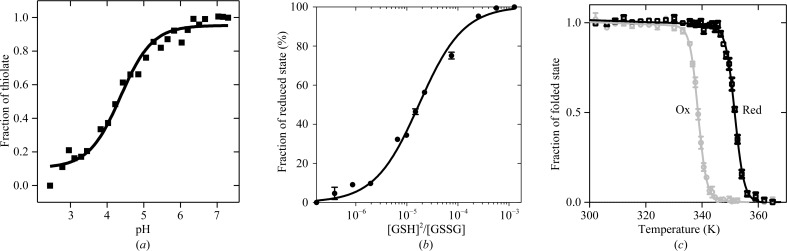
Characterization of the redox properties of MtbDsbA. (*a*) p*K*_a_ determination of the nucleophilic cysteine of MtbDsbA. This is a representative plot of three independent measurements of the pH-dependent thiolate-specific absorbance of the catalytic cysteine. The p*K*_a_ was obtained from the nonlinear fit to the Henderson–Hasselbach equation. (*b*) Determination of the redox equilibria of MtbDsbA with glutathione at pH 7.0 and 298 K. The plot shows the averaged fraction (three replicates) of reduced MtbDsbA at various ratios of reduced:oxidized glutathione. The resulting equilibrium constant *K*_eq_ (17.37 ± 0.1 µ*M*) from the nonlinear curve fit for a one-site binding equation was used to calculate the redox potential of MtbDsbA relative to the glutathione (GSH/GSSG) standard potential of −240 mV (Gilbert, 1995 ▶). (*c*) Relative thermal stability of oxidized (grey) and reduced (black) MtbDsbA at pH 7.0. The normalized average far-UV CD signal from three measurements was fitted to a two-state unfolding model as described previously (Kurz *et al.*, 2009 ▶). The resulting melting temperature (*T*_m_) of MtbDsbA shows that its reduced form (351.7 ± 0.1 K) is more stable than its oxidized form (338.6 ± 0.1 K).

Typically, DsbA enzymes have a highly acidic catalytic cysteine and a highly oxidizing redox potential that enable its participation in thiol–disulfide exchange reactions. To study the redox characteristics of MtbDsbA, we created a double noncatalytic cysteine mutant MtbDsbA^m^ (Cys140Ala, Cys192Ala). Similar to Wpα-DsbA1, this mutation did not affect the solubility and integrity of MtbDsbA. Using a pH-titration experiment (Fig. 1 ▶ *a*), we found that the measured p*K*_a_ of the catalytic cysteine Cys89 is 4.2 ± 0.2, which falls between the previously reported values for EcDsbA (p*K*_a_ = 3.3) and *Vibrio cholerae* DsbA (VcDsbA, p*K*_a_ = 5.1). The redox potentials of DsbA proteins also vary among characterized DsbAs; the most reducing and oxidizing DsbAs reported to date are Wpα-DsbA1 (−163 mV) and neisserial NmDsbA3 (−80 mV), respectively. We measured the standard redox potential by equilibration of MtbDsbA^m^ with glutathione at 298 K and pH 7.0 (Fig. 1 ▶ *b*). We found that the oxidizing power of MtbDsbA (−99 mV) is comparable to that of *Pseudomonas aeruginosa* DsbA (PdDsbA) (−94 mV; Shouldice *et al.*, 2010 ▶), somewhat more oxidizing than EcDsbA (−122 m*M*; Wunderlich & Glockshuber, 1993 ▶) and a little less oxidizing than BsDsbA (−80 m*M*; Crow *et al.*, 2009 ▶).

Another characteristic feature common to DsbA enzymes is that the catalytic cysteine disulfide bond reduces the stability of the protein (Heras *et al.*, 2008 ▶). A thermal unfolding experiment comparing the stability of the redox forms showed that the oxidized (disulfide) form of MtbDsbA is less stable than its reduced form by 13 K (Fig. 1 ▶ *c*), which is in agreement with the findings for other DsbA enzymes.

Taken together, these results show that the putative DsbA protein encoded by *M. tuberculosis* has the redox hallmarks of a DsbA disulfide oxidoreductase.

### 3.3. MtbDsbA is a disulfide oxidase

**Figure 2:**
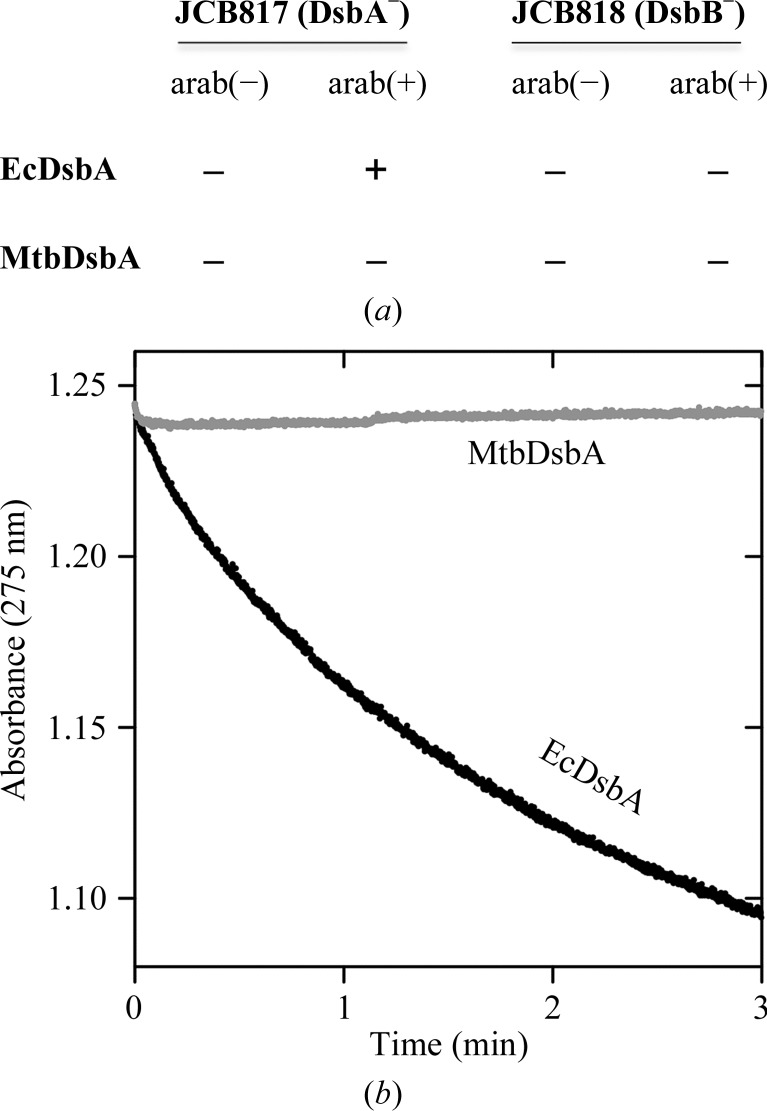
Ability of MtbDsbA to recognize EcDsbB. (*a*) Restoration of *E. coli* motility. Constructs expressing MtbDsbA or EcDsbA (control) were transformed into *E. coli* DsbA null (JCB817) and DsbA/DsbB double null (JCB818) mutant cells. FlgI function is impaired in the absence of EcDsbA or EcDsbB owing to the absence of disulfide-bonding activity (Dailey & Berg, 1993 ▶). The ability to recognize EcDsbB and EcDsbA substrates *in vivo* by MtbDsbA was evaluated by restoration of *E. coli* motility in the agar, as seen in the induced EcDsbA control. Shown is the summary of three replicates of induced (containing arabinose) and uninduced bacterial swarming plates (not containing arabinose, as a negative control). See Supplementary Fig. S2 for bacterial plate images. (*b*) Ubiquinone reduction of EcDsbB–UQ1 by MtbDsbA. The data presented here are the normalized mean absorbance of UQ1 from three independent measurements. EcDsbB was added to the EcDsbA/UQ1 mixture to initiate the reaction.

An *in vivo* complementation experiment has been developed to demonstrate that the expression of a DsbA enzyme can rescue the motility of *E. coli* cells deficient in EcDsbA (Kurz *et al.*, 2009 ▶; Paxman *et al.*, 2009 ▶). However, MtbDsbA does not restore the motility of *E. coli* DsbA null cells as shown by the results of the cell-swarming assay (Fig. 2 ▶ *a*). To establish whether this might be a consequence of a lack of interaction between MtbDsbA and EcDsbB (the essential redox partner of EcDsbA), we monitored the ability of EcDsbB to oxidize MtbDsbA in an EcDsbB-catalyzed ubiquinone reduction assay (Fig. 2 ▶ *b*). This experiment showed as expected that EcDsbB can oxidize EcDsbA, but it failed to redox couple with MtbDsbA. This could explain why MtbDsbA does not complement EcDsbA in the motility assay, but we cannot rule out the possibility that MtbDsbA is in­active because it also does not recognize the P-ring protein (FlgI) substrate of EcDsbA in the assay (Dailey & Berg, 1993 ▶).

We then set out to determine the redox-activity profile of MtbDsbA in standard disulfide oxidase, reductase and isomerase assays.

**Figure 3:**
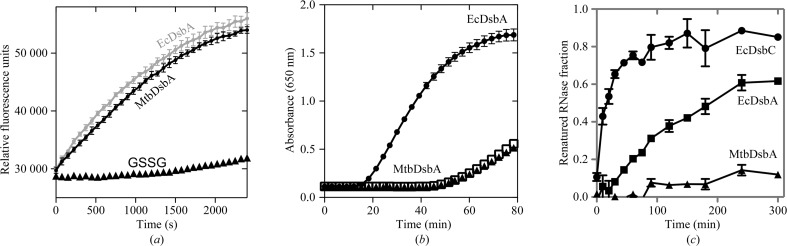
Disulfide oxidoreductase activities. (*a*) Disulfide oxidase activity. Representative fluorescence curves of peptide cysteine oxidation by MtbDsbA and EcDsbA in the presence of glutathione as the electron donor. Enzyme-catalyzed peptide oxidation is significantly faster than the glutathione-mediated reaction. Peptide oxidation in the buffer control or by the catalytically inactive MtbDsbA (Cys89Ala) or EcDsbA (Cys33Ala) was insignificant over the duration of the assay (not shown for clarity). (*b*) Insulin disulfide-reduction assay. The precipitation of insulin by MtbDsbA or EcDsbA or DTT (trace overlaps that of EcDsbA) was monitored as described in §[Sec sec2]2. (*c*) Scrambled RNase disulfide isomerization assay. Disulfide isomerization activity of MtbDsbA, EcDsbA and EcDsbC was monitored using scrambled RNase as the substrate.

The disulfide oxidase activity of MtbDsbA was investigated using an *in vitro* peptide-oxidation assay with a fluorescently labelled peptide substrate (CQQGFDGTQNSCK), using GSSG as the electron donor to regenerate the oxidized DsbA (Fig. 3 ▶ *a*). MtbDsbA catalyzed peptide oxidation well above the background rate [in the absence of enzyme or in the presence of the catalytic cysteine mutant MtbDsbA (Cys92Ala)]. However, EcDsbA-mediated peptide oxidation was found to be faster than MtbDsbA as it required five times more MtbDsbA to match the catalytic rate of EcDsbA. This suggests the possibility that MtbDsbA and EcDsbA recognize the substrate peptide with differing affinities or kinetics.

The reductase activity of MtbDsbA was assessed spectrophotometrically by analyzing the ability of the enzyme to reduce the interchain disulfides of insulin (measured as an increase in turbidity through precipitation of the insulin B chain; Fig. 3 ▶ *c*). In contrast to EcDsbA, MtbDsbA did not show any disulfide reductase activity against insulin, supporting the possibility that the two enzymes have different substrate-binding surfaces.

Finally, we tested the ability of MtbDsbA to isomerize, or shuffle, the incorrect disulfides of scrambled RNaseA to form the correctly folded RNaseA. Under the conditions of the assay, MtbDsbA was able to generate just 10% of the activity of fully folded RNaseA after 5 h (Fig. 3 ▶ *c*). By comparison, EcDsbA and EcDsbC (a disulfide isomerase enzyme) produced ∼60 and ∼90%, respectively, of the activity of correctly folded RNase over the same time period. These observations are consistent with the view that MtbDsbA has the characteristic redox properties of a disulfide oxidase but differs functionally in other redox assays compared with the prototypical EcDsbA oxidase.

### 3.4. The crystal structure of MtbDsbA reveals unique features

**Figure 4:**
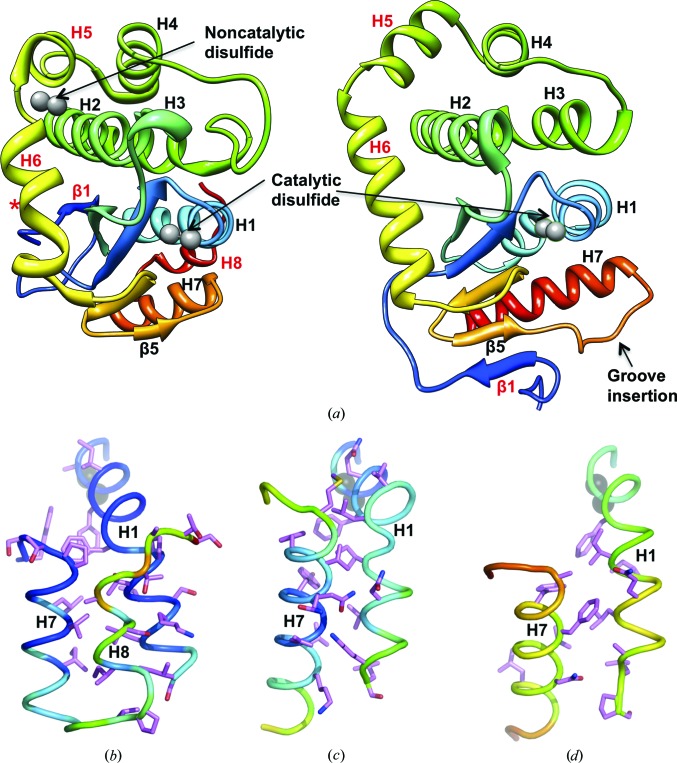
Structural comparison of MtbDsbA. (*a*) The crystal structure of MtbDsbA (left) is compared with the prototypical EcDsbA (right; PDB entry 1fvk; Guddat *et al.*, 1998 ▶). Catalytic/noncatalytic cysteine residues are shown as light grey spheres. The noncatalytic structural disulfide of MtbDsbA is absent in EcDsbA. Helix H8 appears to be unique to MtbDsbA. The orientation of helix H5 dramatically varies in these two proteins. Gly204 breaks the hydrogen-bonding pattern in the middle of helix H6 of MtbDsbA (helix H6 is kinked in MtbDsbA compared with EcDsbA). MtbDsbA appears to be much smaller in size than EcDsbA in this orientation. However, the molecular surface areas of MtbDsbA (8208 Å^2^) and EcDsbA (8670 Å^2^) are comparable. The intramolecular interaction of helix H1 with the C-terminal region of (*b*) MtbDsbA, (*c*) EcDsbA (PDB entry 1fvk; Guddat *et al.*, 1998 ▶) and (*d*) VcDsbA (PDB entry 1bed; Hu *et al.*, 1997 ▶) is also shown. For (*b*), (*c*) and (*d*) the backbone colour is set to the temperature factor from the PDB. The side chains of interfacing residues identified by *PISA* (*Protein Interfaces, Surfaces and Assemblies*; Krissinel & Henrick, 2007 ▶) analysis are shown as sticks. Catalytic and noncatalytic cysteines are shown as grey spheres.

To explore the atomic details of MtbDsbA, we determined its structure at 2.0 Å resolution. The structure was solved by MR searches using trimmed ‘Poly Ser’ templates derived from remote DsbA homologues (see §[Sec sec2]2 and Supplementary Fig. S1). Two independent MtbDsbA molecules were well resolved in the asymmetric unit (residues 57–255; 199 residues for each monomer) and were refined to *R*_work_ and *R*_free_ values of 14.5 and 19.2%, respectively (Table 1 ▶). The MtbDsbA structure has a canonical DsbA-like architecture comprising a Trx domain with a helical domain inserted into the middle of the Trx domain (Fig. 4 ▶ *a*). The two MtbDsbA molecules in the asymmetric unit are very similar (r.m.s. deviation of 0.26 Å for all C^α^ atoms). However, the structure of MtbDsbA differs significantly from the prototypical EcDsbA with an r.m.s. deviation of 3.7 Å for 149 C^α^ atoms (PDB entry 1fvk chain *A*; 3.7 Å for 148 C^α^ atoms for 1fvk chain *B*; Guddat *et al.*, 1997 ▶). Notable differences from EcDsbA include the folding topology of the central β-sheet, an additional helix H8 at the C-terminus and a much shorter connecting loop between β5 and H7 (Fig. 4 ▶ *a*) that forms part of the binding interface between EcDsbA and its redox partner EcDsbB. As in all thioredoxin-like proteins, the catalytic C*XX*C motif is located at the N-­terminus of helix H1 in MtbDsbA.

A structural comparison of MtbDsbA against all structures in the PDB using *DALI* (Holm *et al.*, 2008 ▶) revealed similarities to other DsbA structures, albeit with relatively large r.m.s. deviations (2.1–4.0 Å for 148–168 C^α^ atoms) and weak sequence conservation (Supplementary Fig. S3). The closest structural similarity occurs with DsbAs from Gram-positive organisms: BsDsbA (r.m.s. deviation 2.6 Å for 168 C^α^ atoms; 21% identity) and SaDsbA (r.m.s. deviation 2.1 Å for 154 C^α^ atoms; 21% identity). The central four strands of the core β-sheet have the same topology (3–2–4–5) in the Trx domains of this group of DsbAs. However, a topological difference is noted in the arrangement of strand β1 (Figs. 4 ▶ and 5 ▶ *a*): in MtbDsbA, BsDsbA and WpDsbA1 β1 forms hydrogen bonds to β5 (3–2–4–5–­1), whereas in the majority of DsbA structures β1 forms hydrogen bonds to β3 on the opposite edge of the β-sheet (1–­3–2–4–5).

A unique feature of MtbDsbA compared with all other structurally characterized DsbAs is the C-terminal extension that forms an additional helix, H8, that packs against helices H1 and H7 (Figs. 4 ▶ *b* and 5 ▶ *a*). Crystallo­graphic *B* factors plotted onto these helices suggest the possibility that the H8 helix may reduce the mobility of the catalytic helix H1 in comparison to EcDsbA and VcDsbA (Figs. 4 ▶ *b*–4*d* ▶). We then analyzed the interface between helix H1 and the C-terminal H8 region (Table 3 ▶). Compared with other structurally characterized DsbAs, the presence of helix H8 in MtbDsbA buries a greater proportion of helix H1 (buried surface area of 592 *versus* 401–260 Å^2^). The H8 helix interaction may restrict the conformational flexibility of helices H1 and H7. Similarly, the second disulfide bond (Cys140–Cys192) of MtbDsbA that links helices H2 and H5 may also restrict flexibility of the four-helix bundle (Figs. 4 ▶ and 5 ▶ *a*). The functional effects of these conformational restrictions are unclear but might explain at least in part the differences that we observe in the functional assays for MtbDsbA and EcDsbA.

**Figure 5:**
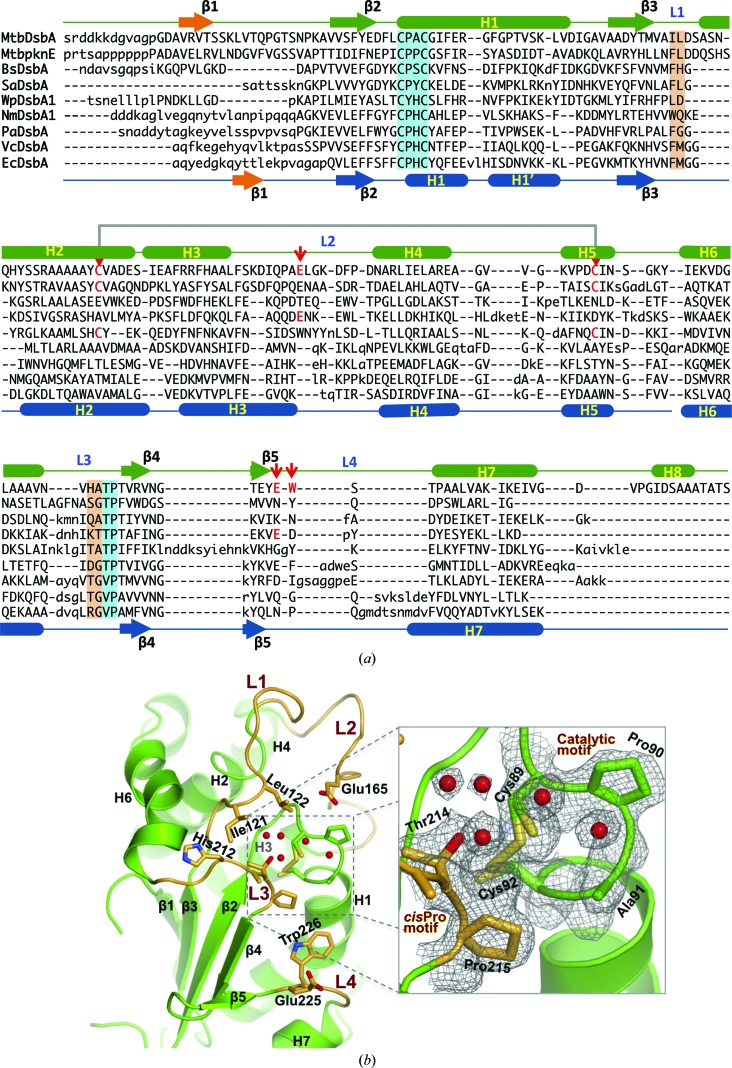
Structural elements of DsbAs and the catalytic site of MtbDsbA. (*a*) Structure-based sequence alignment of DsbAs. Structurally equivalent positions (upper case), variable regions (lower case) and insertions (dashes) are shown. PknE DsbA sequence alignment is based on MtbDsbA and BsDsbA (see Supplementary Fig. S6 for the PknE hypothetical model). Secondary-structure assignments for MtbDsbA (top green), EcDsbA (bottom blue) and topological variations originating at strand β1 in MtbDsbA and EcDsbA (orange) are presented. The catalytic motif and *cis*-Pro motif are highlighted in cyan and the equivalent EcDsbA residues involved in partner/substrate interaction are highlighted in orange. A negatively charged residue (Glu165) in the vicinity of the catalytic cysteine, a residue blocking the hydrophobic groove (Trp226) and a noncatalytic structural disulfide bond are marked with red arrows (see text for details). (*b*) The structure of the catalytic face of MtbDsbA. Residues forming the putative binding surface and negatively charged residues neighbouring the catalytic cysteine are shown. The inset displays the 2*mF*_o_− *DF*_c_ electron-density map around the catalytic motif, the *cis*-Pro loop and ordered active-site water molecules (1.0σ contour level).

The highly conserved structural regions in these DsbAs are the catalytic motif and *cis*-proline (*cis*-Pro) loop, both of which are known to be critical for activity in other DsbA enzymes (Kadokura *et al.*, 2004 ▶). However, the catalytic motif (Cys89-Pro90-Ala91-Cys92) in MtbDsbA differs from other structurally characterized DsbAs, which are generally Cys-Pro-His/Tyr/Ser-Cys. The catalytic cysteines Cys89 and Cys92 display alternative side-chain conformations corresponding to oxidized and reduced states (estimated approximately 50 and 65% for reduced states on the basis of crystallographic occupancy), despite the fact that oxidized protein was used for crystal growth (Fig. 5 ▶ *b*). The active-site disulfides of DsbA proteins are susceptible to radiation damage in the crystal (Guddat *et al.*, 1998 ▶) and the broken disulfide bond in MtbDsbA is therefore likely to be a result of the synchrotron radiation used to measure the diffraction data. In its reduced form in the crystal, Cys89 of MtbDsbA is likely to exist as the thiolate (p*K*_a_ 4.2, pH in the crystal drop 5.5). Ordered active-site water molecules are present in the crystal structure at positions consistent with formation of stabilizing hydrogen bonds to the Cys89 thiolate and to Thr214, a residue preceding the *cis*-Pro loop. This hydrogen-bonding network differs from that in EcDsbA, which has a Val at the equivalent position to Thr214, but is similar to that in SaDsbA, which also has a Thr prior to the *cis*-Pro residue (Heras *et al.*, 2008 ▶).

### 3.5. The surface surrounding the active site of MtbDsbA differs from that of EcDsbA

**Figure 6:**
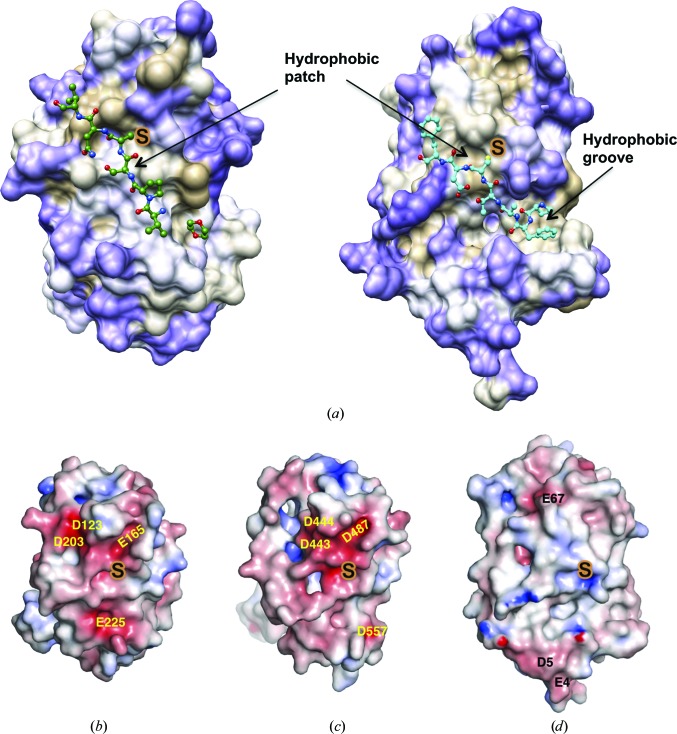
Comparison of surface properties. (*a*) Hydrophobicity surface presentations for MtbDsbA and EcDsbA. Surface colourings are mapped to the Kyte–Doolittle hydrophobicity scale from purple (most hydrophilic) to white to tan (most hydrophobic). The MtbDsbA structure is shown (left) with the modelled VKOR peptide (VPSCNV) and crystallographically identified artificial ligand 1,4-dioxane (see text and Supplementary Fig. S4 for details). The EcDsbA–EcDsbB complex structure is shown on the right (PDB entry 3e9j; Malojcić *et al.*, 2008 ▶). For clarity only the periplasmic loop segment ‘PFATCDF’ of EcDsbB is shown. Electrostatic surface comparisons of (*b*) MtbDsbA, (*c*) the hypothetical model of PknE DsbA and (*d*) EcDsbA. Electrostatic surface potential is contoured between −6 (red) and +6 (blue) *kT*/e. The homology model of PknE (template based on MtbDsbA and BsDsbA) was prepared in *MODELLER* (Eswar *et al.*, 2006 ▶) and atomic clashes were minimized in *Chiron* (Ramachandran *et al.*, 2011 ▶).

The conformation and amino-acid composition of the loop connecting the Trx domain and the helical domain (β3–H2, L1), an inter-helix loop (H3–H4, L2), the *cis*-Pro loop (H6–β4, L3) and the connecting loop β5–H7 (L4) create the surface features surrounding the catalytic cysteine (Cys89) of MtbDsbA (Fig. 5 ▶). Crystal structures of EcDsbA in complex with protein partners have shown that the hydrophobic patch created by loops L1 and L3, the active-site motif and the hydrophobic groove formed by loop L4 and helix H1 contribute to interactions with partner proteins (Paxman *et al.*, 2009 ▶; Inaba *et al.*, 2006 ▶). The length and amino-acid identity of these loops are generally not conserved among DsbAs (Fig. 5 ▶ *a*). The relatively long L4 loop in EcDsbA creates a large hydrophobic groove compared with the short loop found in MtbDsbA, SaDsbA and BsDsbA (Fig. 6 ▶). In MtbDsbA, the side chain of Trp226 (L4 loop) sits in a shallow groove formed by Ile94, Phe95, Gly98, Phe99, Ser227, Thr228 and Pro229 (Fig. 5 ▶ *b*). The hydrophobic nature of the surrounding catalytic residues is largely preserved in MtbDsbA (Fig. 6 ▶). Unexpectedly, a ligand, 1,4-dioxane from the crystallization solution, was bound in this shallow groove, forming contacts with Ile94, Gly98, Phe99 and Pro229 (Supplementary Fig. S4). MtbDsbA also has two negatively charged residues near the catalytic Cys89, similar to those observed in SaDsbA (Glu165 and Glu225, see Fig. 5 ▶ *b*). The SaDsbA glutamate equivalent to Glu165 has been shown to influence the p*K*_a_ of the catalytic cysteine (Heras *et al.*, 2008 ▶).

### 3.6. MtbDsbA binds a peptide derived from VKOR

Although the binding surface of MtbDsbA does not have the distinct binding groove of EcDsbA that interacts with a periplasmic loop of its membrane partner EcDsbB, we investigated whether a fragment of a periplasmic loop from the putative binding partner MtbVKOR would interact with MtbDsbA. We used ITC to assess the binding of MtbDsbA to several peptides derived from MtbVKOR (see Supplementary Fig. S5 for the quality of the ITC data). Topological prediction indicated that MtbVKOR is comprised of five transmembrane helices connected by two periplasmic loops. Cys57 from the first periplasmic loop has been shown to form a mixed disulfide bond with EcDsbA (Wang *et al.*, 2011 ▶), although a direct interaction of MtbVKOR with any mycobacterial protein has not been reported. We found that the MtbVKOR 11-residue peptide segment surrounding Cys57 (^51^PIYVPS­CNVNP^61^) binds to MtbDsbA with an apparent *K*_d_ of 3.8 µ*M* (Table 4 ▶). Trimming from either end of this peptide showed that a minimal hexapeptide (^54^VPSCNV^59^) binds to MtbDsbA with *K*_d,app_ = 6.7 µ*M*. Further, modelling of the ^54^VPSCNV^59^ peptide onto MtbDsbA suggested that Tyr53 of the VKOR sequence might bind to the site occupied by 1,4-dioxane in the crystal structure (Fig. 6 ▶ *a*). Indeed, this heptapeptide ^53^YVPSCNV^59^ showed improved binding (*K*_d,app_ =2.9 µ*M*) to MtbDsbA. Binding of this VKOR peptide to MtbDsbA is cysteine-dependent, as substitution of Cys with Ala or Leu or mutation of the MtbDsbA catalytic Cys89 to Ala abolished binding under the conditions tested.

### 3.7. Comparison with the predicted mycobacterial DsbA-like domain of PknE

Aside from MtbDsbA, the H37Rv strain encodes five other putative extracytoplasmic Trx-related proteins: DsbE (Rv2878c), DsbF (Rv1677), Rv3673c, Rv0526 and the transmembrane protein kinase PknE (Rv1743). PknE is a clear structural homologue of MtbDsbA and also contains a kinase domain. Curiously, sequence searches suggest that PknE orthologues are restricted to pathogenic mycobacteria and PknE appears to be the only Mtb protein kinase fused to a DsbA homologous domain. The PknE predicted DsbA domain (residues 377–566) is linked to the intracellular protein kinase domain (residues 1–289) through a single-pass transmembrane helix (residues 337–359). Sequence comparison and secondary-structure prediction are consistent with the notion that PknE DsbA shares the same overall fold as that of MtbDsbA. We generated a homology model for PknE DsbA based on the MtbDsbA structure to compare the sequence variation in the context of the three-dimensional structure (Supplementary Fig. S6). Predicted differences from MtbDsbA in the PknE DsbA domain include the absence of helix H8 and a short helix H7 at the C-terminus. There are also sequence variations in loops L1, L3 and L4 (Fig. 5 ▶ *a*). Predicted similarities in PknE DsbA include conservation of the Thr-*cis*-Pro motif, a glutamate equivalent of Glu225 and a tyrosine equivalent of Trp226. The calculated electrostatic potential surfaces are negative in the region surrounding the catalytic site in both proteins (Figs. 6 ▶ *c* and 6 ▶ *d*). However, the CPPC catalytic motif in PknE DsbA differs from that of CPAC of MtbDsbA. These differences may contribute to a difference in redox characteristics. In EcDsbA, mutation of CPHC to CPPC dramatically increased its reducing potential (−122 mV to −220 mV; Bessette *et al.*, 2001 ▶). The native CPPC active site in a DsbA homologue from *Salmonella enterica* (SeSrgA) has also been linked to a more strongly reducing potential (−154 mV). Indeed, the insulin reductase activity of SeSrgA was shown to be similar to that of the disulfide isomerase EcDsbC (Heras *et al.*, 2010 ▶).

## 4. Discussion

Novel drug targets are desperately needed to combat tuberculosis. Mycobacterial proteins exported across the cytoplasmic membrane play important roles in Mtb adhesion, invasion, virulence, pathogenesis and survival inside host cells. Drugs targeting export systems in Gram-negative bacteria have been tested for their ability to block delivery of effector proteins required for virulence (Moir *et al.*, 2011 ▶). Likewise, there are intensive efforts to develop antituberculosis drugs that target the major mycobacterial protein-export systems: the Sec pathway, the Tat pathway and the ESX pathways (Feltcher *et al.*, 2010 ▶; McCann *et al.*, 2011 ▶).

Another approach is to target the disulfide pathways that mediate disulfide-bond formation and correct folding of exported proteins in Mtb (Chim *et al.*, 2010 ▶, 2011 ▶; Goulding *et al.*, 2004 ▶). In bacteria, disulfide-bond formation in newly exported proteins is commonly mediated through mechanisms that rely on electron transfer between redox proteins. The best-studied protein redox pair, DsbA/DsbB, is found in most aerobic α-, β- and γ-proteobacteria. However, in mycobacteria and other actinobacteria, cyanobacteria and δ- and ∊-proteobacteria, it has been proposed that DsbA/VKOR forms a functional redox pair to catalyze disulfide-bond formation (Dutton *et al.*, 2008 ▶; Li *et al.*, 2010 ▶; Wang *et al.*, 2011 ▶). The currently accepted mechanism is that the cysteine-containing periplasmic loop of transmembrane protein DsbB or VKOR is transiently drawn into the binding groove of DsbA (Fig. 6 ▶) to drive the flow of electrons (Inaba & Ito, 2008 ▶). Oxidized DsbA then exchanges its catalytic disulfide bond with newly exported proteins by transiently interacting with them through its catalytic cysteine and binding surface (Inaba & Ito, 2008 ▶; Inaba *et al.*, 2006 ▶; Kadokura *et al.*, 2003 ▶). Thus, compounds targeting the protein–protein interaction surface responsible for interaction between DsbA and its substrate proteins or between DsbA and DsbB/VKOR may inhibit virulence-factor maturation in pathogens.

The data we present here show that Rv2969c has disulfide oxidase activity, a highly acidic active-site cysteine, a destabilizing active-site disulfide and a highly oxidizing potential. Structurally, MtbDsbA has a canonical DsbA architecture with a C*XX*C active-site motif. We therefore conclude that Mtb Rv2969c is an authentic DsbA enzyme. Our data also show that MtbDsbA is able to interact tightly with a seven-residue peptide corresponding to the periplasmic loop of MtbVKOR and these data support the notion that MtbDsbA and MtbVKOR are a functional redox pair that could catalyze disulfide-bond formation in newly translocated substrates.

Helix H8 at the C-terminus of MtbDsbA and its interaction with active-site helix H1 are unique to MtbDsbA among structurally characterized DsbAs. This feature appears to be absent in the DsbA-like domain of PknE. Substrate binding and mobility of DsbAs has been linked to the catalytic activity of DsbA (Horne *et al.*, 2007 ▶). In this context, restraint of helix H1 by helix H8 in MtbDsbA may contribute to the catalytic activity of MtbDsbA. Moreover, the relatively negative surface potential surrounding the catalytic site of MtbDsbA compared with EcDsbA suggests that these two enzymes have very different substrate-binding specificities. This is supported by our assay data showing the relative activities of these two enzymes in assays for reductase (MtbDsbA no activity), oxidase (MtbDsbA slower activity) and isomerase (MtbDsbA marginal activity) activity. EcDsbA is known to be a promiscuous enzyme that interacts with many substrates. Our structural and functional data suggest that MtbDsbA may have a more limited number of specific substrates.

Previous work has shown that the anticoagulant warfarin that inhibits human VKOR can also inhibit MtbVKOR and it interferes with the growth of Mtb in cell studies (Dutton *et al.*, 2010 ▶). Inhibiting MtbVKOR is an appealing strategy for treating Mtb, but will require the design of selective inhibitors that target MtbVKOR and not its human counterpart. Our results suggest that MtbDsbA may represent an alternative target for inhibiting disulfide oxidation in Mtb and for preventing the production of virulence factors.

## Supplementary Material

PDB reference: Rv2969c, 4k6x

Supplementary material file. DOI: 10.1107/S0907444913017800/tz5033sup1.pdf

## Figures and Tables

**Table 1:** Summary of data-collection and refinement statistics Values in parentheses are for the highest resolution shell.

Space group	*I*4_1_22
Unit-cell parameters (Å)	*a* = 92.05, *b* = 92.05, *c* = 232.76
Data-collection wavelength (Å)	0.9537
Resolution range (Å)	85.6–1.97 (2.08–1.97)
No. of observations	286100 (39641)
No. of unique reflections	35185 (5080)
Mean *I*/σ(*I*)	14.9 (3.3)
*R*_merge_	0.096 (0.66)
*R*_p.i.m._	0.036 (0.246)
Multiplicity	8.1 (7.8)
Wilson *B* factor (Å^2^)	26.1
Model and refinement statistics	
Resolution range (Å)	59.3–1.97
No. of unique reflections	35175
Completeness	98.4
*R*_work_ (%)	14.5 (20.3)
*R*_free_ † (%)	19.2 (28.4)
No. of non-H atoms	
Protein	2996
Ligand	17
Water	415
R.m.s.d., bond lengths (Å)	0.011
R.m.s.d., bond angles (°)	1.27
Ramachandran favoured/allowed (%)	97.5/99.5
Average *B* factor (Å^2^)	
Protein	23.7
Ligand	24.6
Water	34.6

† *R*_free_ is calculated as for *R*_work_ but for 5% of the total reflections chosen at random and omitted from refinement.

**Table 2:** Survey of Trx-related proteins in *M. tuberculosis* strain H37Rv Summary of the 15 Trx-related proteins found using keyword and *BLASTP* searches. Only Rv2969c and Rv1743 could be considered similar to DsbA-like proteins. Structural similarities to Trx-related proteins were identified using *BLASTP* and *Fold and Function Assignment* (*FFAS*; http://ffas.burnham.org/) searches against PDB codes or % identity to reported structure (PDB code in parentheses) are given. The functional annotation, presence and location of signal peptide/membrane-spanning region and operon are derived information from Target TB annotation (http://genome.tbdb.org/). The presence of signal sequence and transmembrane region were also confirmed using *SignalP*3.0 and transmembrane prediction using hidden Markov models (*TMHMM*). DsbA-like (Rv2969c) is predicted to have an N-terminal transmembrane (TM) helix or signal peptide (SP).

Protein	Length	Functional annotation	Signal peptide	Structural representative	Operon
PknE-DsbA (Rv1743)	566	Ser/Thr kinase	Integral membrane	2h34 (kinase; Gay *et al.*, 2006 ▶), 31% (DsbA-like)	Ser-Thr protein kinase, fused DsbA-like
DsbA-like (Rv2969c)	255	Conserved, unknown	TM or SP	Present work	Pyruvate carboxylase-like, VKOR, Dsb-like
DsbE (Rv2878c)	173	Unknown	Yes	1lu4 (Goulding *et al.*, 2004 ▶)	Rv2877c, DsbE
DsbF (Rv1677)	182	Unknown	Yes	3ios (Chim *et al.*, 2010 ▶)	Rv1676, DsbF
ResA-like (Rv3673c)	227	Unknown	Yes	30% (DsbE/F)	Rv3673c, endonuclease III
Trx-related (Rv0526)	216	Unknown	Yes	47% (3lwa; Midwest Center for Structural Genomics, unpublished work)	HemeL, Rv0525, Rv0526, ccdA, Rv0528, ccsA
TrxC (Rv3914)	116	Disulfide exchange	No	3o6t (Hall *et al.*, 2011 ▶)	TrxB2, TrxC
TrxB2 (Rv3913)	335	Disulfide reductase	No	2a87 (Akif *et al.*, 2005 ▶)	TrxB2, TrxC
TrxB1 (Rv1471)	123	Disulfide exchange	No	36% (3hhv; Ruggiero *et al.*, 2009 ▶)	TrxA, TrxB1
TrxA (Rv1470)	124	Disulfide exchange	No	32% (3hhv; Ruggiero *et al.*, 2009 ▶)	TrxA, TrxB1
Trx-like (Rv0816c)	140	Disulfide exchange	??	28% (3ilu; Ptak *et al.*, 2009 ▶)	Rv0816c, Rv0817c
Trx-like (Rv2183c)	131	Conserved, unknown	??	14% (3raz; New York SGX Research Center for Structural Genomics, unpublished work)	Rv2183c, Rv2184c
Trx-fusion protein (Rv1324)	304	Disulfide exchange	No	24% (3qdn; Center for Structural Genomics of Infectious Diseases, unpublished work)	Rv1324
Trx-related (Rv2286c)	230	Conserved, unknown	No	20% (3fz5; Midwest Center for Structural Genomics, unpublished work)	Rv2286c
Trx-related (Rv2466c)	207	Unknown	No	14% (3fz5; Midwest Center for Structural Genomics, unpublished work)	Rv2466c

**Table 3:** Analysis of the interface between helix H1 and the C-terminal region of DsbA structures The buried surface area (BSA) in helix H1 (column 3) was determined using *PISA* analysis (Krissinel & Henrick, 2007 ▶). DsbAs, their PDB codes (column 1, in parentheses) and the residue ranges in helix H1 (column 2, in parentheses) and the C-terminal region (column 4, in parentheses) included in the *PISA* analysis are listed. The percentage of surface area buried in helix H1 relative to its total accessible surface area (∼2000 Å^2^) is given in column 3 (in parentheses). For MtbDsbA and EcDsbA, BSA and the values reported for the percentage of surface area buried are an average of two MtbDsbA chains (molecule 1 and molecule 2 in the asymmetric unit) and nine EcDsbA chains [PDB entries 1dsb (two chains; Martin *et al.*, 1993 ▶), 1fvk (two chains; Guddat *et al.*, 1997 ▶), 1a2m (two chains; Guddat *et al.*, 1998 ▶), 1a2l (two chains; Guddat *et al.*, 1998 ▶) and 1a2j (one chain; Guddat *et al.*, 1998 ▶)].

Protein (PDB code)	No. of interfacing residues in helix H1	Helix H1 BSA (Å^2^)	No. of interfacing residues in C-terminal region
MtbDsbA	13 (89–109)	592 ± 4 (29.6)	18 (226–255)
EcDsbA	9 (30–50)	401 ± 42 (18.9)	10 (168–188)
BsDsbA (3eu3; Crow *et al.*, 2009 ▶)	8 (69–91)	355 (15.9)	8 (205–222)
SaDsbA (3bci; Heras *et al.*, 2008 ▶)	9 (26–46)	370 (15.8)	7 (167–178)
PaDsbA (3h93; Shouldice *et al.*, 2010 ▶)	7 (37–55)	282 (14.3)	9 (166–192)
VcDsbA (1bed; Hu *et al.*, 1997 ▶)	8 (30–48)	261 (13.7)	6 (164–181)
WpDsbA (3f4r; Kurz *et al.*, 2009 ▶)	9 (51–71)	363 (16.5)	9 (196–218)
NmDsbA1 (3a3t; Vivian *et al.*, 2009 ▶)	7 (57–74)	290 (15.5)	7 (189–212)

**Table 4:** Thermodynamic parameters for VKOR-derived peptides binding to MtbDsbA *K*_d,app_, apparent dissociation constant; Δ*H*, enthalpy change; *T*Δ*S*, temperature (K) × entropy change; *N* is the apparent stoichiometry. *T*Δ*S* is calculated from the free energy equation (Δ*H* − *T*Δ*S* = −*RT*ln*K*_d,app_). Values reported are the mean and standard deviation of the curve fits from two ITC runs in each case. Representative ITC profiles are provided in Supplementary Fig. S5. n.b., no binding detected. 1 cal = 4.184 J.

	*N*	*K*_d,app_ (µ*M*)	Δ*H* (kcal mol^−1^)	−*T*Δ*S* (kcal mol^−1^)
PIYVPSCNVNP	1.0	3.8 ± 0.2	−10.9 ± 0.0	3.5
VPSCNVNP	1.0	5.9 ± 2.6	−7.1 ± 4.0	2.5
VPSCNV	1.0	6.7 ± 0.0	−6.8 ± 0.2	−0.1
VPSLNV		n.b.		
YVPSCNV	1.1	2.9 ± 0.3	−7.0 ± 0.4	−0.5
YVPSANV		n.b.		

## Footnotes

1 Supplementary material has been deposited in the IUCr electronic archive (Reference: TZ5033). Services for accessing this material are described at the back of the journal.
