# Supplementary material for: Rv2969c, essential for optimal growth in Mycobacterium tuberculosis, is a DsbA-like enzyme that interacts with VKOR-derived peptides and has atypical features of DsbA-like disulfide oxidases
Source: Acta Crystallogr D Biol Crystallogr. 2013 Sep 20;69(Pt 10):1981–94. doi: 10.1107/S0907444913017800 (PMC3792642; doi:10.1107/S0907444913017800)

Supplementary Material

Rv2969c, essential for optimal growth in *Mycobacterium tuberculosis,* is a DsbA-like enzyme that interacts with VKOR-derived peptides and has atypical features of DsbA-like disulfide oxidases

Lakshmanane Premkumar[a]*, Begoña Heras[ab], Wilko Duprez[a], Patricia Walden[a], Maria Halili[a], Fabian Kurth[a], David P. Fairlie[a] and Jennifer L. Martin[a]*

[a]Institute for Molecular Bioscience, Division of Chemistry and Structural Biology, University of Queensland, St Lucia, QLD, 4067, Australia
[b]Current address: Latrobe Institute for Molecular Sciences, Latrobe University, Melbourne, NSW, Australia

Correspondence email: j.martin@imb.uq.edu.au; p.lakshmanane@imb.uq.edu.au

Supplementary Figure S1

Molecular replacement phasing of MtbDsbA with a ‘twilight zone’ template. (A) Amino acid sequence alignment. SaDsbA (3bci) shares 18% sequence identity and 83% coverage to MtbDsbA. However, successful MR solution could only be obtained using a trimmed “Poly-Ser” template derived from SaDsbA that shares 48% sequence identity and just 39% coverage to MtbDsbA. Identical residues are shown in red. See Figure S3 for structure-based sequence identities and RMSDs of MtbDsbA to other DsbAs. (B) Structural overlays of MtbDsbA (blue), SaDsbA (green) and “poly-Ser” template (brown).

A

```
MtbDsbA      SRDDKKDGVAGPGDAVRVTSSKLVTQPGTSNPKAVVSFYEDFLCPACGIFERGFGPTVSKLVD
SaDsbA       ......................ASATTSSKNGKPLVVVYGDYKCPYCKELDEKVMPKLRKYID
MR-template  .................................ASVSSYSDSSCPACGSSSSGSGPSSSKSSD

IGAVAADYTMVAILDSASNQHYSSRAAAAAYCVADESIEAFRRFHAALFSKDIQPAELGKDFPDNARLIELAREA
NHKVEYQFVNLAFLGKD-----SIVGSRASHAVLMYAPKSFLDFQKQLFAAQQDENKEW---LTKELLDKHIKQL
SGAVAASSSSSA...........SSAAAAASSVASSSSSAFSSFSAALF................ASLSSSASS.

GVVGKVPDCINSGKYIEKVDGLAAAVNVHATPTVRVNGTEYEWSTPAALVAKIKEIVGDVPGIDSAAATATS
HLDETENKIIKDYKTKDSKKKIAKDNHIKTTPTAFINGEKVEDPYDYESYEKLLKDKIKLE...........
...............................PTSSSN...................................
```

B

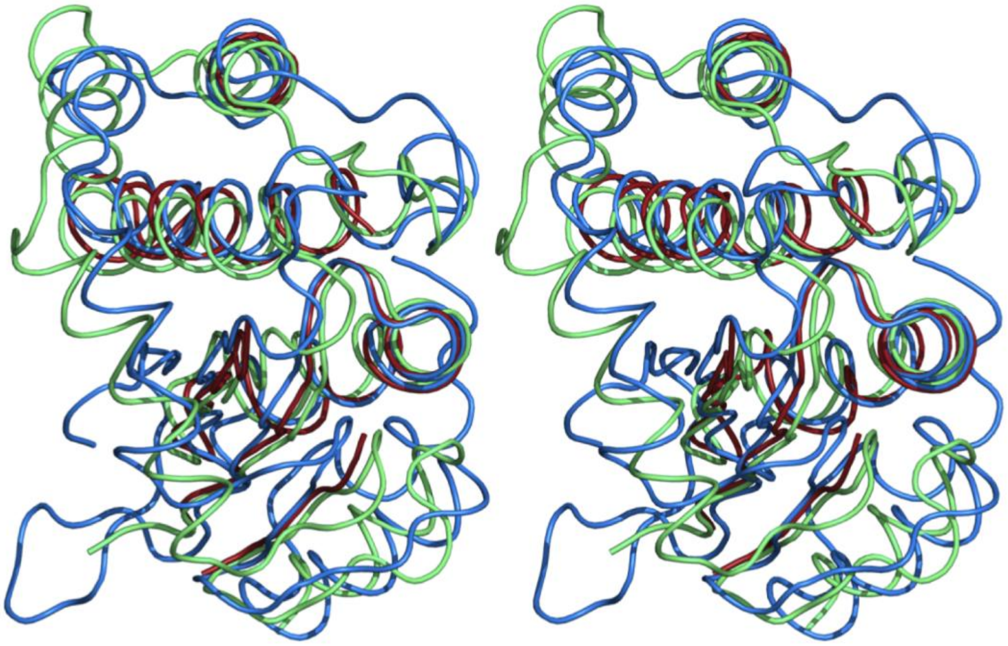

Supplementary Figure S2

EcDsbA *in vivo* complementation assay. EcDsbA or MtbDsbA was cloned into a EcDsBA signal sequence under an arabinose inducible promotor. As shown, MtDsbA does not complement EcDsbA in dsbA null JCB817 cells. As expected, EcDsbA rescues the motility of JCB817 cells, but not DsbA/DsbB double null JCB818 cells, in an arabinose dependent manner. See text for details.

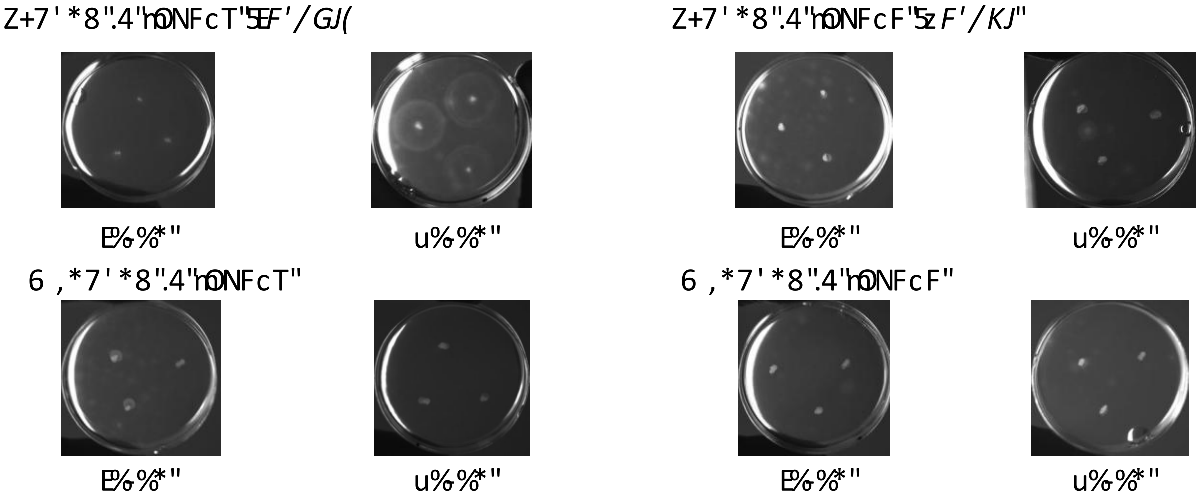

Supplementary Figure S3

Assessment of structural homologs for MtbDsbA by DALI. The top structural homologs with significant sequence coverage are listed. “Z-scores” above 6 are considered significant. Columns are: PDB code, “Z-score”, RMS deviation on Cα for the given number of aligned residues (align), the total number of residues in the protein are shown under aa. %id is the % identity of the aligned residues. Gram-positive organisms are highlighted in green.

PDB Id	Z-Score	RMSD	align	aa	%id	Protein
3eu3-A	19.4	2.6	168	186	21	*Bacillus subtilis BDBD*
3bci-A	17.7	2.1	154	165	21	*Staphylococcus aureus DsbA*
3f4r-A	15.5	2.9	163	199	17	*Wolbachia pipientis DsbA1*
3gyk-A	14.7	3.2	156	174	19	*Silicibacter pomeroyi DSS-3 DsbA-like*
1z6m-A	14.2	2.8	150	175	13	*Enterococcus faecalis DsbA-like*
3gn3-A	13.3	3.3	155	179	14	*Pseudomonas syringae DsbA-like*
3dvw-A	12.1	3.5	151	191	16	*Neisseria meningitidis DsbA1*
3dvx-B	11.4	3.4	149	186	15	*Neisseria meningitidis DsbA3*
1bed-A	11.1	3.9	150	181	16	*Vibrio cholerae TcpG*
3h93-A	11.3	4.0	152	192	18	*Pseudomonas aeruginosa DsbA*
2rem-B	11.3	3.9	150	187	14	*Xylella fastidiosa DsbA-like*
1fvk-A	11.0	3.7	149	188	16	*Escherichia coli DsbA*

Supplementary Figure S4

Crystallographic identification of the binding of an artificial ligand 1,4-dioxane in MtbDsbA. 1,4-dioxane (DIO) from the crystallization solution was bound in a shallow sub-pocket contributed by the catalytic helix H1 and loop L4. Residues surrounding 1,4-dioxane are shown. A simulated annealing omit difference map for 1,4-dioxane is shown contoured at 3.0 σ.

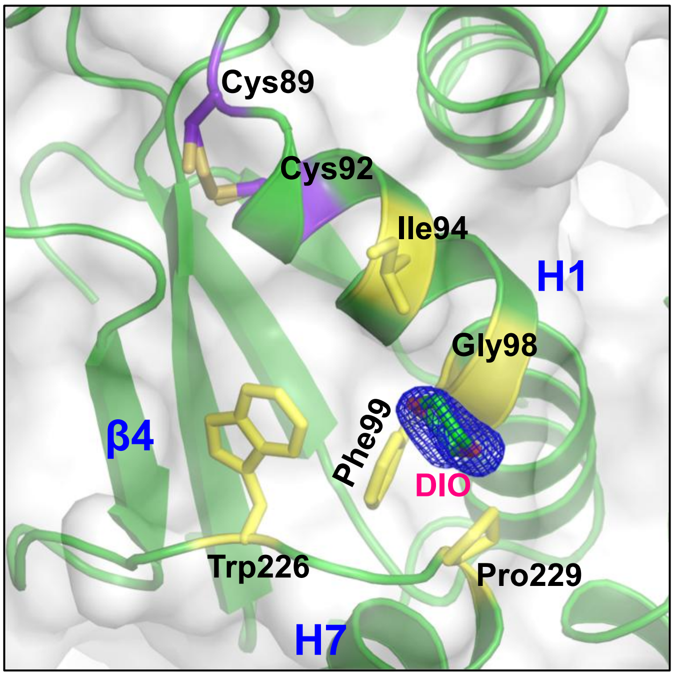

Supplementary Figure S5

Representative isothermal titration calorimetry profiles for VKOR-derived peptide binding to MtbDsbA. The data were generated using 2 µl injections of peptides at a concentration of 2-4 mM titrated into a cell containing 100 µM MtbDsbA.

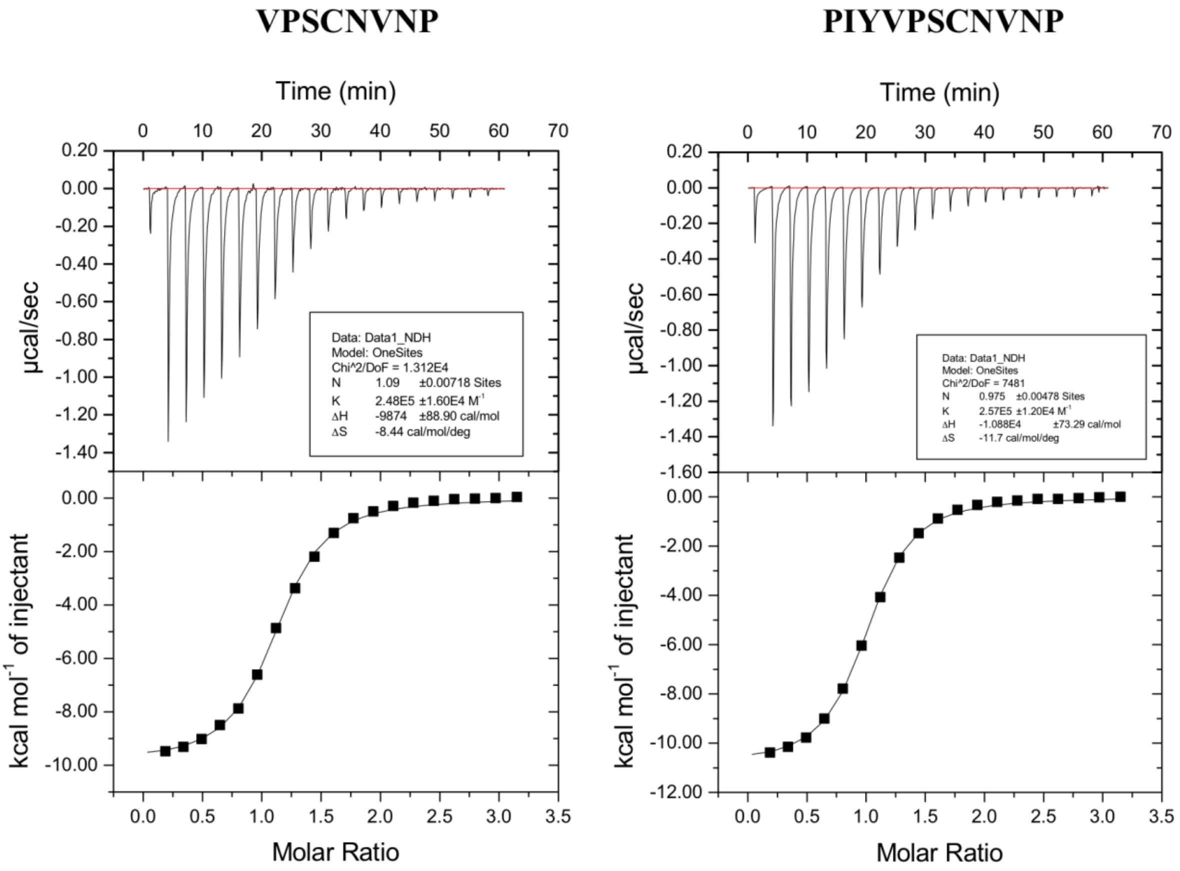

Supplementary Figure S6

Structural comparison of MtbDsbA crystal structure and modelled structure of PknE DsbA domain. Homology model of PknE (template MtbDsbA and BsDsbA) was prepared in Modeller (Eswar *et al.*, 2006) and atomic clashes were minimized in Chiron (Ramachandran *et al.*, 2011). Spheres show the position of cysteines forming the catalytic and non-catalytic disulfides. The non-catalytic disulfides are marked with an asterisk.

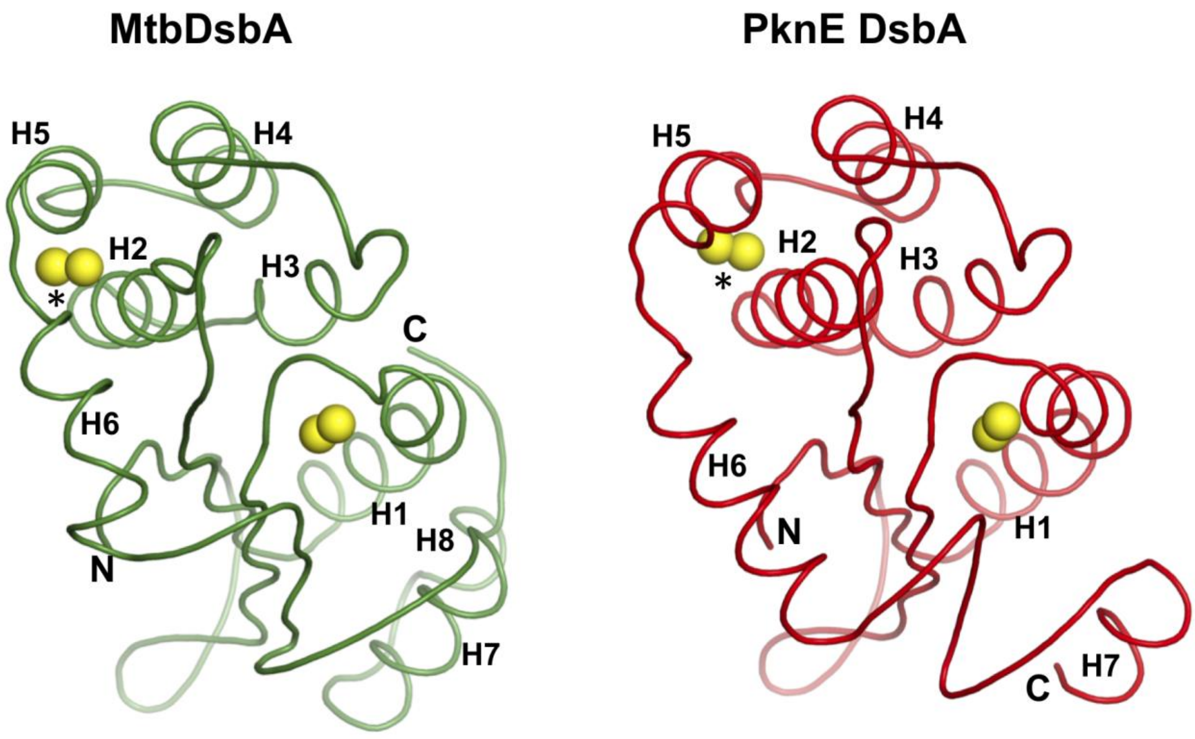

Supplement: Supplementary file 1 [file d-69-01981-sup1.pdf]
